# Factors associated with withdrawal of life-sustaining therapy in severe traumatic brain injury patients

**DOI:** 10.1186/cc9940

**Published:** 2011-03-11

**Authors:** N Côte, A Turgeon, F Lauzier, L Moore, JF Simard, D Scales, K Burns, M Meade, F Bernard, D Zygun, D Fergusson

**Affiliations:** 1 Université Laval, Québec, Canada; 2 University of Toronto, Canada; 3 McMaster University, Hamilton, Canada; 4 Université de Montréal, Canada; 5 University of Calgary, Canada; 6 University of Ottawa, Canada

## Introduction

Traumatic brain injury (TBI) mortality remains high and often follows withdrawal of life-sustaining therapy (WLST). Studies reporting the determinants of WLST in this population are scarce. We analyzed data from a multicenter retrospective cohort study to identify factors associated with WLST in TBI.

## Methods

We randomly selected charts of 720 mechanically ventilated severe TBI patients (identified using ICD-10 codes) admitted to the ICUs of six participating centers (120 patients per center) over a 2-year period. Data were abstracted using a standardized case report form and operations manual. Among nonsurvivors (*n *= 228), we compared patients who died following WLST with those who did not in order to investigate the potential influence of variables pertaining to the injury and management. Our final model to WLST included four baseline characteristics (age, gender, GCS and pupillary reflex) and factors with *P *< 0.2. Research ethics approval was obtained in all participating centers.

## Results

We analyzed 225 patients (three missing data) including predominantly male patients (69.7%) with a mean age of 50.7 years. Among nonsurvivors, brain herniation on initial CT scan was more often reported in patients dying following WLST (OR = 2.91, 95% CI = 1.16 to 7.30, *P *= 0.02), while the opposite was observed for epidural hematoma (OR = 0.18, 95% CI = 0.06 to 0.56, *P *< 0.01). Craniotomy (OR = 0.12, 95% CI = 0.02 to 0.68, *P *= 0.02) and other non-neurosurgical procedures (OR = 0.08, 95% CI = 0.02 to 0.43, *P *< 0.01) were associated with a lower odds of death following WLST. Other interventions, such as vasopressor use (OR = 0.50, 95% CI = 0.22 to 1.11, *P *= 0.09), DVT prophylaxis (OR = 0.33, 95% CI = 0.11 to 1.03, *P *= 0.06) and insulin infusions (OR = 2.13, 95% CI = 0.99 to 4.62, *P *= 0.06) were not significantly associated with lower and higher odds of death due to WLST, respectively.

## Conclusions

Death due to WLST was associated with several patient and clinical factors. We also observed that WLST was less frequent among patients that had received more aggressive treatments, for example craniotomy. Further research is required to understand factors that influence decisions to WLST in severe TBI patients, since these decisions may be modifiable and based on physicians' and surrogates' perceptions of prognosis.

